# Growth Performance and Characterization of Meat Quality of Broiler Chickens Supplemented with Betaine and Antioxidants under Cyclic Heat Stress

**DOI:** 10.3390/antiox8090336

**Published:** 2019-08-22

**Authors:** Majid Shakeri, Jeremy J. Cottrell, Stuart Wilkinson, Hieu H. Le, Hafiz A. R. Suleria, Robyn D. Warner, Frank R. Dunshea

**Affiliations:** 1 Faculty of Veterinary and Agricultural Sciences, The University of Melbourne, Parkville, VIC 3010, Australia; 2 Feedworks Pty Ltd., Romsey, VIC 3434, Australia

**Keywords:** heat stress, betaine, antioxidants, growth performance, meat quality, broiler chickens

## Abstract

Heat stress (HS) causes oxidative stress, which compromises broiler performance and meat quality. The aim of this study was to determine whether dietary antioxidants could be used as an amelioration strategy. Seventy-two day-old-male Ross-308 chicks were exposed to either thermoneutral or cyclical heat stress conditions. Diets were either control commercial diet (CON), CON plus betaine (BET), or with a combination of betaine, selenized yeast, and vitamin E (BET + AOX). Heat stress increased the rectal temperature (*p* < 0.001), respiration rate (*p* < 0.001), decreased blood pCO_2_ (*p* = 0.002), and increased blood pH (*p* = 0.02), which indicated the HS broilers had respiratory alkalosis. Final body weight was decreased by HS (*p* < 0.001), whereas it was improved with BET (*p* = 0.05). Heat stress reduced cooking loss (*p* = 0.007) and no effect on drip loss, while BET decreased the drip loss (*p* = 0.01). Heat stress reduced the myofibril fragmentation index (*p* < 0.001) and increased thiobarbituric acid reactive substances (*p* < 0.001), while these were improved with the combination of BET + AOX (*p* = 0.003). In conclusion, BET overall improved growth rates and product quality in this small university study, whereas some additional benefits were provided by AOX on product quality in both TN and HS broilers.

## 1. Introduction

The management of heat stress (HS) is a subject of increasing concern for industry with increasing global temperatures and incidence of sub-tropical and tropical broiler production [1]. Broilers are susceptible to increased environmental heat loads for a variety of reasons, including that they lack sweat glands, feather coverage, and that increased selection for muscling means they are comparatively less resilient to heat than other production animals [2]. Broilers that are reared under hot conditions have a reduced growth rate, in part due reduced feed intake. However, only half of the consequential reduction in growth rates is due to feed intake alone [3], and skeletal muscle from thermally challenged broilers has reduced rates of protein synthesis and to a lesser extend proteolysis [4]. This is, in part, due to adaptive endocrine alterations to HS, for example, thyroid hormones are permissive for growth and negatively correlated with elevated temperature. Furthermore, insulin is an important regulator of muscle metabolism and protein synthesis, and the downstream anabolic and metabolic pathways of insulin signaling are suppressed in the skeletal muscle of the HS broiler [5]. Other implications for product quality include reduced muscle glycogen and muscle pH, paler colour [6], increased lipid oxidation [7], and changes muscle fiber structure [8,9]. Consequently, there is a growing interest in affordable dietary strategies to reduce the adverse effects of HS on meat quality and performance in domestic animals [10,11]. Some of the most promising strategies include betaine (BET), selenium (Se), and vitamin E (VitE), which have individually shown positive effects on chicken performance and meat quality during HS.

Betaine is a natural compound that can be found in many organisms and it is commercially derived from sugar beets. It protects cells against osmotic inactivation, increases water retention of cells [12], and improves protein and energy metabolism [13] by acting as a methyl donor, which is important for the synthesis of methionine and carnitine. Betaine is a potent antioxidant [14] and dietary BET can increase plasma glutathione peroxidase in chickens under both thermoneutral (TN) and HS conditions [15]. It also reduces the core body temperature by reducing the activity of the ion pumps that are required for osmoregulation, allowing more energy for growth [16,17,18]. In this regard, BET may improve body weight and meat quality in chickens [19,20]. Selenium is an antioxidant that may affect growth performance by the maintenance of tissue cellular integrity [21,22]. The activity of Se is often augmented through interactions with VitE, which maximizes the efficiency of the vitamin as an antioxidant. There are indications that Se deficiency has deleterious effects on growth performance and meat quality [23,24]. Moreover, VitE improves performance and meat quality by protecting and regenerating damaged tissues during oxidative stress [25,26] and reducing lipid peroxidation and drip loss [27].

In a previous investigation on the individual and combined effects of BET and the antioxidants Se and VitE (AOX) on growth performance and physiological response to HS we observed improved performance in response to BET alone, and no positive effects of AOX [15]. However, we did see some additional benefits of AOX when combined with BET. Therefore, the aims of the present study were to investigate the effects of BET alone and in combination with Se and VitE on the growth performance and meat quality characterization and the physiological responses to HS in broiler chickens.

## 2. Materials and Methods

### 2.1. Ethics Statement

The study was performed in Animal facility approved by the Animal Ethics Committee of the Faculty of Veterinary and Agricultural Sciences, The University of Melbourne, Australia (Protocol no. 1714224.1). The use of animals for research is regulated by federal and state legislation, according to the Prevention of Cruelty to Animals Act 1986 and the Prevention of Cruelty to Animals Regulations 2008.

### 2.2. Animals, Diet and Experimental Design

Seventy-two one day-old male Ross-308 chicks were obtained from a commercial hatchery and transported at ~30 °C within 2 h distance to our facility (Tri Foods Pty. Ltd., Bannockburn, Victoria, Australia). The chicks were randomly selected, wing-tagged, and individually weighed and housed in pens of three chicks in one of two environmentally controlled rooms (12 pens/room). The temperature for both rooms was held constant at 33 °C for the first seven days of the study before being gradually decreased to 25 °C from days 7 to 21 for the TN room (1 °C decrease every two days), and then remained at 25 °C from days 21 to 42 [28]; or, cyclic HS conditions where the temperature was kept at 33 °C from days 7 to 42 between 9 am and 5 pm and at the prevailing temperature in the TN room from 5 pm until 9 am. The relative humidity was between 45–60 % during the study. Light was provided 24 h for the first three days after placement and it was gradually reduced (1 h/day) to 20 h at day 7 for both rooms. The pens measured 1 m × 1 m and they had ~10 cm deep wood shavings on the floor.

The chickens were fed either a commercial control diet (50 IU/kg VitE and 0.3 mg/kg Se) (CON), or the control diet supplemented with 1 g/kg betaine (Betafin S1, DuPont, Marlborough, UK) (BET) or the control diet plus a combination of 1 g/kg betaine with 0.3 mg/kg Se (selenised yeast, selenoSource 3,000, Diamond V Mills, Cedar Rapids, IO, USA) and 200 IU/kg natural yeast VitE (VE 200, dl-α-tocopheryl acetate, ADM, Chicago, IL, USA) (BET + AOX). The control diet was formulated to satisfy the NRC [29] requirements with Se provided as selenised yeast and the VitE as natural VitE, as outlined above. Feed (Feedworks BESTMIX, The University of Sydney, Sydney, Australia) was formulated as a commercial starter from days 1 to 14 (CP 24.7 % and 12.6 MJ ME/kg), grower from days 15–28 (CP 22.8 % and 13.0 MJ ME/kg) and finisher from days 29–42 (CP 21.24 % and 13.4 MJ ME/kg). Feed and water were provided *ad libitum*. Live weight and consumed feed were recorded weekly to calculate the average daily body weight gain (ADG), average daily feed intake (ADFI), and feed conversion ratio (FCR).

Starting from day 14, two chickens per pen were randomly selected weekly to measure rectal temperature at 8 am, 12 pm, and 15 pm with a digital thermometer (Comark PDT 300, Norwich, Norfolk, UK) inserted about 3–4 cm into the rectum for 30 sec. The respiration rate was measured from two randomly selected chickens per pen at 12 pm on days 35 and 41. Chickens were filmed with a cellphone (iPhone 7, Apple Inc., Cupertino, CA, USA) and then the number of breaths taken over a 20 sec. period was quantified and then expressed as breaths per minute.

Twenty-four chickens were randomly selected to collect blood samples (four from each treatment) on day 41. Approximately 3 mL blood was collected from the wing vein and centrifuged at 10,000× *g* for 15 min to obtain plasma. A portion (~0.3 mL) of the fresh blood sample was loaded into an automatic blood gas analysis (EPOC^®^; Alere, Waltham, MA, USA) to measure blood gas and chemistry parameters.

### 2.3. Slaughter and Meat Quality Measures

All of the chickens were killed with a dry electrical stunner (standard 15 amp 220-volt, Mitchell Engineering Food Equipment, Pty Ltd., Clontarf, Queensland, Australia). Subsequently, the chickens were immersed in hot water of 60–62 °C for 80 s. before they were mechanically stripped of the feathers (1.5 Kw, 660 mm, Bellsouth Poultry Equipment, Pty Ltd., Dandenong, South Victoria, Australia). All of the carcasses were then placed in sealable plastic bags and sealed before being suspended in ice water (~4 °C) for 40 min and the carcasses were then transferred to a cool room (4 °C). These slaughter procedures follow standard industry practice. The *m. Pectoralis major and minor*, hereon referred to as the breast muscle, were removed from the carcass at 24 h after slaughter.

Muscle pH was measured with a polypropylene spear type electrode (Ionode IJ44A, Pty Ltd., Tennyson Queensland, Australia), calibrated at pH 4 and 7 and portable pH meter (WP-80M, TPS, Pty Ltd., Brendale, Queensland, Australia) by inserting ~2 cm deep for 30 s. Immediately after the removal of the feathers, the breast muscles were partially skinned and pH measured three times at >10 min., 1 h and 24 h after slaughter before deboning. Drip loss was quantified by weighing 10 g of breast meat and then suspending in a bottle with a sealed cap for 48 h. All of the samples had freshly trimmed surfaces and they were devoid of covering fat or connective tissue. After 48 h, all of the samples were re-weighed to calculate drip loss. Warner Bratzler shear force (WBSF) was quantified on blocks of cooked meat 1 cm wide, 1 cm high, and 3 cm lengthwise (Instron, canton, MA, USA). Prior to cooking, samples (80 g) were weighed and were then placed in a plastic bag and then cooked in an 80 °C water bath for 40 min. After cooking, the samples were cooled in running tap water for 10 min., and then gently dried with a piece of paper towel without being squeezed to remove all moisture lost on the meat surface before they were re-weighed to measure cooking loss. The cooked samples were chilled overnight at 4 °C to measure WBSF after 24 h. Cooked samples were cut into blocks and WBSF was measured using an inverted V-blade and cross head speed 200 mm/min. perpendicular to muscle fiber orientation and Lloyd (Lloyd Instruments LS5S/C Materials testing instrument, Lloyd Instruments Ltd., Hampshire, UK) texture analyser fitted with a 500 N load cell. Warner Bratzler shear force was performed at least six times for each sample. A Minolta chromameter CR-400 with 8 mm aperture (Minolta Pty Ltd., Tokyo Japan, light source D65, observer angle 2°) was used to determine the colour at days 1 and 4 post-slaughter after a 20 min. bloom at 4 °C with measurements in the CIE L*, a*, b* system where L* indicates relative lightness, a* redness and b* yellowness. Breast muscle was placed on retail trays, over-wrapped, and then placed in simulated retail display, with illumination, at 4 °C for three days before measuring colour. All of the readings were taken in triplicate and averaged.

The method of [30], as modified by [31], was used with some modifications to measure myofibrillar fragmentation index (MFI). Briefly, 2 g minced breast meat was added to 20 mL MFI buffer (100 mM KCl, 20 mM potassium phosphate (pH 7), 1 mM EGTA, 1 mM MgCl_2_, and 1 mM NaN_3_) and then homogenized for 30 s. at 10,000× *g* (IKA Ultra Turrax ^®^ T 25 digital, Rawang, Selangor, Malaysia). All samples were kept in crushed ice during homogenizing. Samples were centrifuged at 100× *g* for 15 min. at 2 °C. After centrifuging, supernatants were discarded and 20 mL MFI buffer was added to each tube. Once more, the samples were centrifuged for another 15 min., at 100× *g* at 2 °C. Subsequently, the supernatant and fat cap were discarded. 10 mL MFI was added to each tube and vortexed until well mixed. Samples were filtered through a polyethylene cell strainer (100 µm). Protein assay was done by adding 0.25 mL suspension with 0.75 mL MFI buffer and 4 mL biuret reagent (1.5 g CuSO_4_·5H_2_O, 6g NaKC_4_H_4_O_6_·4H_2_O, 300 mL 10% NaOH adding to 700 mL dH_2_O). The mixture was kept in a dark place at room temperature for 30 min. Simultaneously, bovin serum albumin was prepared as 0, 2.5, 5, 7.5, and 10 mg/mL. The absorbance of prepared standards was read at 540 nM with a spectrophotometer (UV-1800, Shimadzu, Kyoto, Japan). The myofibril protein concentration of the samples was adjusted to 0.5 mg/mL and then all of the prepared samples were vortexed and mixed well prior to reading absorbance with spectrophotometer at 540 nM (UV-1800, Shimadzu, Kyoto, Japan).

Thiobarbituric acid reactive substances (TBARS) were measured as per [32], with minor modifications. To determine TBARS, 2 g of breast muscles were homogenized in 8 mL potassium chloride with homogenizer (IKA Ultra Turrax ^®^ T 25 digital, Rawang, Selangor, Malaysia) for 30 s. at 10,000× *g*. Afterwards, 200 µL of homogenized samples was mixed with 35 µL butylated hydroxy toluene 7.0 mM, 165 µL 8.1% sodium dodecyl sulphate, 2 mL thiobarbituric acid 0.8%, topped up with 300 µL distilled water. Subsequently, the mixture was heated at 95 °C for 60 min. The mixture was cooled under running water for 5 min., and 3 mL n-butyl alcohol was added into the mixture, and then vortexed for 60 s. The mixture was centrifuged at 3000× *g* for 10 min. and the supernatant A_532_ quantified *vs* a malondialdehyde (MDA) (mg/kg) standard curve (UV-1800, Shimadzu, Kyoto, Japan).

### 2.4. High Performance Liqiud Chromatography Analysis of Betaine and Derivatives

Betaine concentrations were determined in liver, muscle, and plasma samples. Standards and samples were prepared while using the method of [33], with some modifications. The frozen tissue samples were first pulverized in a dry mill pre-cooled in liquid nitrogen (The Cellcrusher, Cork, Ireland). Pulverized tissue (0.5 g) was homogenized with homogenizer (IKA Ultra Turrax ^®^ T 25 digital, Rawang, Selangor, Malaysia) for 1 min. at 10,000× *g* in 5 mL tris buffer (1 M, pH 7), centrifuged at 14,000× *g* for 20 min., and the supernatant was then collected. The supernatant and plasma samples (50 μL) was added to 50 μL of 100 mmol/L monopotassium phosphate and 900 μL derivatization solution containing 50 mmol/L 4-bromophenacyl bromide and 2.5 mmol/L 18-crown-6 in acetonitrile and vortex mixed. The samples were heated to 80 °C in a block heater for 1 h, cooled to room temperature before filtering through a 0.22 µm filter into a glass High Performance Liquid Chromatography (HPLC) vial, and then placed on a wisp model 712 autosampler attached to a HPLC. The HPLC (Waters 2998, Milford, MA, USA), comprising a quaternary pump, vacuum degasser, an autosampler, and a column compartment with thermostat and a photodiode array (PDA) detector, with data acquisition from Empower software (Water, Milford, MA, USA). A XBrigge columns (HILIC 3.5 µm, 4.6 × 150 mm, Waters, Milford, MA, USA) was used, with the temperature being maintained at 25 °C. An isocratic mobile phase solution contained 22 mmol/L choline chloride (Sigma-Aldrich, St. Louis, MI, USA) in 900 mL/L acetonitrile and 100 mL/L distilled water were used at a flow rate of 1.0 mL/min. The mobile phases were filtered by using a 0.45 µm filter and then degassed by sonication for 30 min. before use. The injection volume for the analysis of all samples was 10 µL. The UV detection was set to monitor the analytes at 254 nm.

### 2.5. Plasma Thyroid Hormone Analysis

The thyroid hormones were measured and validated by using free triiodothyronine (T_3_) and Thyroxine (T_4_) radioimmunoassay kit in plasma (MP Biomedicals, LLC Diagnostics Division, Fountain Parkway, Solon, OH, USA). For both T_3_ and T_4_, plasma samples were kept at room temperature before use in order to minimise deterioration. For T_3_, 100 µL of standards and samples were added to each tube. Then, 1 mL free T_3_ tracer was added to each tube and vortexed for 3–5 s. before being incubated in a water bath for 150 min. at 37 °C. After incubating, the liquid was aspirated or decanted from all tubes. Added 1 mL distilled water to each tube and aspirated again. All of the tubes were read with a gamma counter (PerkinElmer Life Science, Wallac 1470 Wizard gamma counter, Waltham, MA, USA). The obtained results were calculated based on the structure that was provided by the company and the results are presented as pg/mL for T_3_. All of the procedures were the same for T_4_, except 50 µL of standards and samples were used, the incubation time was 90 min., and the results were presented as ng/dL.

### 2.6. Statistical Analyses

All of the data were analysed while using ANOVA for the main and interactive effects of temperature, pooled BET (Control *vs* BET and BET + AOX), and within BET (BET *vs* BET + AOX) using GenStat version 16^th^ (VSNi Ltd., Hemel Hempstead, UK). These planned contrasts allows for the separation of the response to BET as well as any additional responses to AOX [34]. For physiological parameters and meat pH and color data the effects of main and interactive effects of time were also included as repeated measures effects in the residual maximum likelihood model. These data were separately analysed due to the large increase in the magnitude and variation in TBARS between 0.5 h and 24 h post slaughter. Differences were considered a trend when 0.10 > *p* > 0.05 and significant when *p* < 0.05.

## 3. Results

### 3.1. Growth Performance

From days 1–21, there were no effects of temperature or dietary treatments on average daily gain (ADG), average daily feed intake (ADFI), and feed conversion ratio (FCR) (Table 1). Between days 21 and 42, there was a reduction in ADG (96.7 *vs* 85.9 g/d, *p* < 0.001) and ADFI (188 *vs* 172 g/d, *p* = 0.017), but no effect on FCR in response to HS. BET tended to increase ADG between days 21 and 42 (88.3 *vs* 92.9 g/d, *p* = 0.10), but it had no effect on ADFI or FCR. Over the duration of the entire study there was a reduction in ADG (72.0 *vs* 65.4 g/d, *p* < 0.001) and ADFI (128 *vs* 118 g/d, *p* = 0.033) but no effect on FCR in response to HS. BET tended to increase ADG over the entire study (66.4 vs 69.9 g/d, *p* = 0.056) but had no effect on ADFI or FCR. As expected from the growth performance data, HS reduced final weight (3065 *vs* 2783 g, *p* < 0.001) and breast weight (737 *vs* 633 g, *p* < 0.001). Dietary BET increased final weight (2825 *vs* 2974 g, *p* = 0.052) and breast weight (634 *vs* 710 g, *p* < 0.002). There were no within BET effects of AOX of growth performance or final and breast weight, which indicated that there were no additional or negative effects of additional supplementation with AOX nor were there any significant interactions.

### 3.2. Physiological Responses

**Figure 1:**
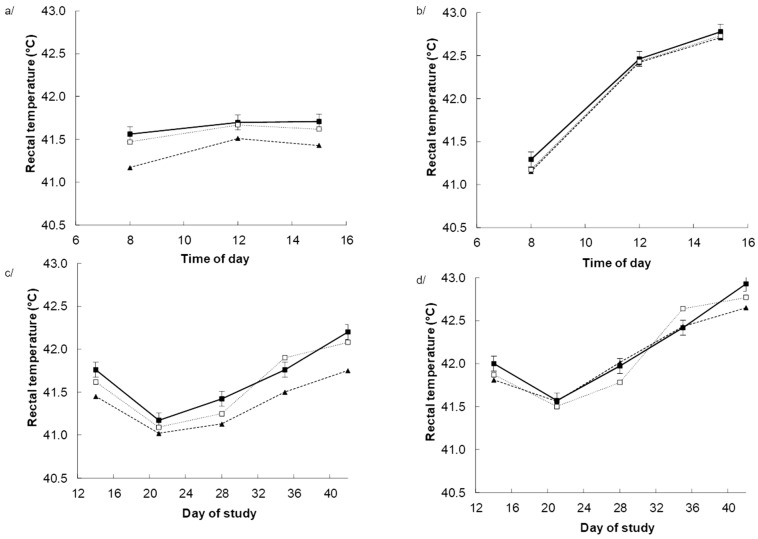
Rectal temperature in chickens fed either a control diet (CON, filled square), the control diet plus betaine (BET, filled triangle) or the control diet plus both BET supplemental antioxidants (BET + AOX, open square) under either thermoneutral (**a**,**c**) or cyclic heat stress (**b**,**d**). Panels (**a**,**b**) indicate the effect of time of day (TM) pooled across days of the experiment with the standard error of the difference for the interaction between TM, BET, and within BET displayed on the data from the chicken receiving the control diet. Panels (**c**,**d**) indicate the effect of day of experiment pooled across time of day with the standard error of the difference for the interaction between day of experiment (DAY), BET, and AOX displayed on the data from the chicken receiving the control diet. There were main effects (*p* < 0.10) of temperature (TEMP) (*p* < 0.001), DAY (*p* < 0.001) and TM (*p* < 0.001), BET (*p* = 0.059), and TEMP × Day (*p* < 0.001). TEMP × TM (*p* < 0.001), DAY × TM (*p* = 0.004), DAYS × BET (*p* = 0.063) TEMP × DAY × TM (*p* < 0.001) and (DAY × within BET) interactive effects. There were no other main or interactive effects (*p* < 0.10).

Heat stress increased the rectal temperature (*p* < 0.001) with the response being the greatest (*p* < 0.001) during the afternoon when the ambient temperature was highest (Figure 1b) and no effects under TN (Figure 1a). Dietary BET tended to reduce rectal temperature in response to dietary BET, particularly towards the end of the experiment, as indicated by the DAY × BET (*p* = 0.063) interaction. The response appeared to be greater for those chickens receiving dietary BET alone, as indicated by DAY × BET × within BET interaction (*p* = 0.004) interactions (Figure 1c,d).

**Figure 2:**
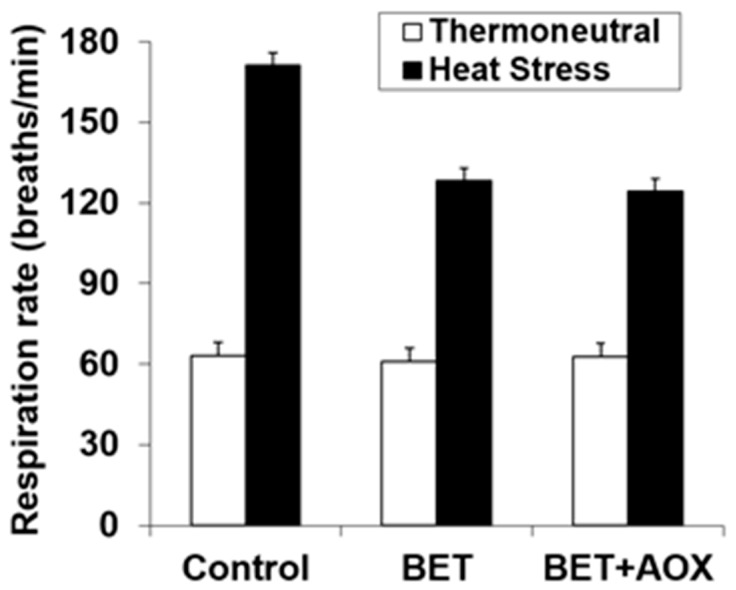
Effect of heat stress and dietary betaine (BET) or BET + AOX (antioxidants) on respiration rate. Since there were no main or interactive effects (*p* > 0.10) of day of study the data are pooled across days 35 and 41 with the standard error of the difference for the interaction between temperature, BET and within BET displayed. There were main effects of temperature (TEMP) (*p* < 0.001) and BET (*p* < 0.001) and a TEMP × BET (*p* < 0.001) interaction. There were no other main or interactive effects (*p* < 0.10).

The respiration rate was increased with HS (62.4 *vs* 141 breaths/min., *p* < 0.001) and it was reduced with BET (118 *vs* 99 breath/min., *p* < 0.001) (Figure 2). However, there was a significant interaction (*p* < 0.001) between HS and BET, such that dietary BET reduced respiration rate during HS (171 *vs* 126 breath/min.), but not under thermoneutral conditions (62.4 *vs* 62.0 breath/min.) (Figure 2). There were no within BET main or interactive effects indicating that there were no effects of additional AOX over that of BET alone.

Blood pH increased during HS (7.31 *vs* 7.39, *p* = 0.021), but it was unchanged by BET (Table 2). Blood partial pressure of CO*_2_* was decreased during HS (53.4 *vs* 41.5 Pa, *p* = 0.002) and unchanged by BET, whereas there was no effect of HS or BET on the partial pressure of O_2_. Blood O_2_ saturation was increased during HS (54.9 *vs* 64.4 %, *p* = 0.029), but it was unchanged by BET (Table 2). There was no effect of HS on total CO_2_, but there were trends towards an effect of BET (*p* = 0.067) and a within BET effect (*p* = 0.058), which showed that the combination of BET and AOX increased the total CO_2_ as compared to both control and BET (26.6 *vs* 27.4 and 30.3 mM for CON, BET, and BET + AOX, respectively). Blood HCO_3_ was decreased during HS (28.1 *vs* 25.8 mM, *p* = 0.024), but it was unchanged by BET. However, there was a within BET effect (*p* = 0.050), such that the combination of BET and AOX increased blood HCO_3_ when compared to both control and BET (26.0 *vs* 26.2 and 28.6 mM). The anion gap tended to be decreased by HS (17.0 *vs* 15.0 mM, *p* = 0.089) and it was decreased by BET (17.8 *vs* 15.1 mM, *p* = 0.039) (Table 2). There was no effect of HS on base excess, whereas BET increased the base excess (−0.74 *vs* 1.09 mM, *p* = 0.047).

Blood haematocrit (21.8 *vs* 18.3 %, *p* = 0.003) and haemoglobin (7.25 *vs* 6.47, *p* = 0.006) decreased during HS, but they were unchanged by BET (Table 3). Blood potassium tended to be decreased by HS (5.99 *vs* 5.62 mM, *p* = 0.071) and decreased by BET (6.05 *vs* 5.68 mM, *p* = 0.092), whereas there was no effect of either HS or BET on sodium. Blood chloride was increased during HS (111 *vs* 114 mM, *p* = 0.014), but it was unchanged by BET. Blood calcium was decreased during HS (1.47 *vs* 1.40 mM, *p* = 0.034), but it was unchanged by BET. Plasma lactate was decreased during HS (7.43 *vs* 4.81 mM, *p* < 0.001) and was decreased (*p* = 0.010) by BET. However, there was also a strong within BET effect (*p* = 0.012), such that the combination of BET and AOX decreased blood lactate to an even greater extent than BET alone (7.35 *vs* 6.53 and 4.48 mM for CON, BET, and BET + AOX, respectively). There was no effect of either HS or BET on blood glucose concentrations. Plasma T_3_ was decreased by HS (*p* = 0.008), while there was no main effect of BET. However, there was a significant interaction between temperature regime and BET (*p* = 0.018), such that plasma T_3_ was increased by BET under TN conditions (4.76 *vs* 6.28 pg/mL) and decreased under HS conditions (5.16 *vs* 3.60 pg/mL). Plasma T_4_ concentrations were decreased during HS (4.57 *vs* 3.23 pg/mL, *p* = 0.049), but it was unchanged by BET.

There were no main or interactive effects of temperature regime on meat colour or pH the data were pooled for presentation (Table 4). Breast muscle L* increased between 24 and 96 h post slaughter (54.6 *vs* 55.2, *p* = 0.011), although there was an interaction between time and BET (*p* = 0.067), such that the L* value increased over time in the breast of those chickens that were fed BET (54.7 *vs* 55.7), but not in those consuming the CON diet (54.3 *vs* 54.2) (Table 4). There were no effects of diet or time on a*, while b* tended to increase between 24 and 96 h post slaughter (1.56 *vs* 1.81, *p* = 0.062). There was the expected decline (*p* < 0.001) in muscle pH over the first 24 h post slaughter. However, there were indications of a within BET (*p* = 0.094) and time × within BET (*p* = 0.10) effects, such that the initial pH was higher in breast muscle from those chickens that were supplemented with both BET and AOX (Table 4).

Cooking loss was decreased in the breast meat from chickens that were exposed to HS (27.3 *vs* 24.9%, *p* = 0.007), while there was no main effect of BET. However, there was an indication of an interaction between temperature regime and BET, such that this response was most pronounced in the breast from chickens fed the CON diet (27.5 *vs* 23.0 %) rather than those consuming BET (27.2 *vs* 25.9 %) (Table 5). While there was no main effect of HS on drip loss there were main (*p* = 0.007) and interactive (*p* = 0.052) effects of BET such that HS increased drip loss in meat from chickens consuming the CON diet (2.11 *vs* 3.43%), but not in those consuming BET (1.76 *vs* 1.69 %). The total water content tended to be lower after HS (75.7 *vs* 75.0 %, *p* = 0.079) but was not significantly altered by BET. There were no main or interactive effects of HS or BET on shear force, although there were indications of a HS × BET (*p* = 0.10) and HS × within BET (*p* = 0.087) interactions that may be attributed to the meat form the chickens consuming the CON diet and housed under TN conditions having the lowest shear force. The MFI was decreased by HS (84.6 *vs* 67.5) but unchanged by BET. However, there was a highly significant BET effect (*p* = 0.003), which, when coupled with the HS × BET interaction (*p* = 0.031), clearly showed that the combination of BET and AOX, but not BET alone, protected against the negative effects of HS on MFI (Table 5). There were no effects of HS or BET on TBARS at 0.5 h post slaughter. However, there were many main and interactive effects at 24 h post-slaughter. Heat stress increased TBARS post slaughter (3.60 *vs* 5.65, *p* < 0.001), whereas TBARS were decreased by BET (5.72 *vs* 4.08, *p* < 0.001). However, there was a within BET effect (*p* = 0.029) and HS × BET (*p* = 0.008) and Hs × within BET (*p* = 0.10) interactions, such that supplemental AOX provided additional antioxidative effects above BET alone under TN conditions, but to a lesser extent under HS conditions.

Plasma BET concentrations were decreased by HS (123 *vs* 112 µmol/L, *p* = 0.043) and increased by BET (94.0 *vs* 130 µmol/L, *p* < 0.001) (Table 6). However, there was a HS x BET interaction (*p* = 0.002), such that HS increased plasma betaine concentrations in chickens consuming the CON diet (85.0 *vs* 102 µmol/L), whereas it was decreased in those consuming BET (143 *vs* 117 µmol/L). There was no main effect of HS on muscle betaine concentrations, whereas it was increased by dietary BET (373 *vs* 456 µmol/g, *p* = 0.009) (Table 6). However, there was a HS × within BET interaction (*p* = 0.050), such that HS decreased muscle betaine concentrations in chickens that were supplemented with BET alone (635 *vs* 518 µmol/g), whereas it was increased in those consuming AOX as well as BET (413 *vs* 567 µmol/g). Liver betaine concentrations were decreased by HS (260 *vs* 165 µmol/g, *p* < 0.001) and decreased by BET (306 *vs* 166 µmol/g, *p* < 0.001) (Table 6). However, there was a HS × BET interaction, such that HS decreased liver betaine concentrations to a greater extent in chickens on the CON diet (403 *vs* 189 µmol/g) whereas it was decreased in those consuming BET (208 *vs* 144 µmol/g). Indeed, chickens consuming the CON diet and housed under TN conditions had the highest liver betaine concentrations. Although statistical comparisons were made, there appeared to be a >1000 fold difference between the plasma and tissue betaine concentrations, suggesting an accumulation against a strong concentration gradient.

## 4. Discussion

The principal findings of this study were that the supplementation of BET alone or in combination with AOX ameliorated symptoms of HS in broilers, as evidenced by reduced respiration rate and rectal temperature. This corresponded to improvements in productive performance, with BET improving the final body and breast weights, irrespective of HS or additional antioxidant supplementation. Betaine supplementation resulted in accumulation within the breast muscle and this corresponded to improved meat quality, namely improved drip loss and lipid stability post-mortem. The temperature profile that was used in this study markedly increased the respiration rate and blood pH, indicating that the broilers were experiencing both HS and respiratory alkalosis. In agreement with other studies, this resulted in an adaptive endocrine state, reducing thyroid hormone concentrations and growth performance [13,15,35,36,37]. Heat stress reduced meat quality, tending to reduce moisture content, increase lipid oxidation, and reduce MFI, a marker of post-mortem myofibrillar breakdown and proteolytic activity. In general, the positive effects of betaine on HS amelioration were not augmented with antioxidant co-supplementation. However, when BET was combined with AOX, there were positive effects on meat quality, reducing postmortem myofibrillar degradation and lipid peroxidation.

Betaine is accumulated in plant and animal tissue when under osmotic stress, and it has previously been reported to accumulate in the hepatic tissue of chicks [38]. Surprisingly, in this study, hepatic BET concentrations were lower with BET and BET + AOX supplementation. The opposite pattern was observed in skeletal muscle, which had higher overall concentrations than the liver. Betaine distribution changed during HS, with concentrations falling in the liver and increasing by ~30%in HS CON chickens. The BET and BET + AOX supplemented chickens further increased their BET concentrations by another 30 %, under TN or HS conditions. As an osmolyte, BET reduces the osmotic gradients, and therefore the activity of cellular ATPases that is required to maintain them [39]. The energy saving from reduced ATPase activity could be substantial, and approximated to be as much as 8% [40]. Although no interactions were observed with circulating thyroid hormones, the reduction of basal metabolic rate has parallels to adaptive endocrine regulation of by thyroid hormones during HS. Triodothyroxine and T_4_ were lower in this study and others [41], and, amongst their biological roles, thyroid hormones serve to set basal metabolic rate though regulating the rate of futile cycling of cellular ATPases. Perhaps part of the reason for the accumulation of betaine in skeletal muscle during HS is thermoregulatory, and a reflection on the physical size of the organ and its contribution to basal metabolic rate. Alternatively, it may reflect accumulation as a protective chaperone.

It has been reported elsewhere that the elimination of betaine is primarily by metabolism and not excretion [42]. Furthermore, the liver is a major site of BET utilization through methyl donor reactions to the methionine cycle via betaine homocysteine methyltransferase (BHMT) and other metabolic pathways. It requires further investigation; however, it is possible that the reductions in hepatic betaine that were observed with supplementation may reflect an increase in utilization. Outside of the DNA methylation reactions, the fates of betaine could be increased remethylation of homocysteine to methionine as part of the methionine cycle. Methionine is an essential amino acid, which is utilized in protein synthesis, as a precursor to cysteine, taurine, and glutathione. If so, this apparent increase in hepatic betaine utilization may explain some of the protective effect of betaine during HS and enhanced growth performance seen in this study and elsewhere [43,44,45]. It has been indicated that the antioxidant properties of betaine provide protection by relieving oxidative pressure and increasing productivity, and the enhancement of meat quality in poultry under stress conditions [46]. During HS, betaine adjusts osmotic pressure, preserves water in cells [43,47], and reduces energy expenditure used in the sodium-potassium pump, which reduces the production of heat from metabolic pathways [48]. By reducing heat production, deep body temperature and respiration rate will decrease, as the chicken’s body does not produce extra heat. Moreover, Se and Vit E are an important part of many metabolic pathways and they enhance immunity, which can improve body temperature management and reduce oxidative damages in cells [49,50]. However, the present results showed no positive effects of Se and VitE on growth performance, which is in line with [50,51]. Indeed, the establishment of Se and VitE requirements are difficult, mainly because of their interactions with diverse factors that may affect the necessity of diet supplementation and the physiological status of the broiler chickens [52].

The present results of blood analysis are in line with a study showing that sodium was unchanged and potassium was decreased with HS [53], while a study reported a reduction of both sodium and potassium [54]. Apparently, the time of blood sampling could be a reason for differences of the results. During HS, hemodilution occurs as the chicken’s body temperature increased due to a reduction of sodium concentration, and some of the tissue potassium exits into the bloodstream apparently due to altered membrane permeability [55]. The results are agreement with studies that showed HS increased blood pH [53,56] and reduced HCO_3_ and chloride, pCO_2_, and haematocrit [53,57]. The increased body temperature that occurs during HS causes broiler chickens to reduce their body temperature through different mechanisms, such as increasing respiration rate. Respiration rate is increased to dissipate heat as well as being a response to the increase in blood pH, owing to respiratory alkalosis. Subsequently, more calcium is needed to exert an acidic action to balance blood pH, resulting in a reduction of HCO_3_. An increased HCO_3_ level in BET + AOX could be related to supplementation of Se that increases calcium release from the sarcoplasmic reticulum [58], which is responsible for storing calcium ions. Respiratory alkalosis causes a primary deficit of CO_2_, and hence causes the ratio of bicarbonate to pCO_2_ to increase, resulting in increased pH. Subsequently, the kidneys attempt to normalize blood pH by increasing the excretion of HCO_3_ by reclaiming H^+^ and by decreasing HCO_3_. Total CO_2_ was higher for BET + AOX that could be attributed to Se level, as it was indicated that higher dosages of Se caused pulmonary congestion and haemorrhages in lungs [59], which could reduce the ability of lungs to reduce the CO_2_ in blood. However, this cannot only be attributed to Se, as the total CO_2_ also increased for BET. This could be related to heavier chickens, there is a negative relationship between surface area available for exchange in lungs and body size [60]. The decreased haematocrit could be related to increased circulating blood volume [61] due to an increase in plasma volume and a lesser increase in the total red blood cells. In broilers, it was observed that the haematocrit decreased when the environmental temperature is higher than 30 °C [62]. Therefore, as hemoglobin is a protein in red blood cells that is responsible for delivery of oxygen, haematocrit reduction results in a reduction of hemoglobin [63]. Unexpectedly, lactate in blood was reduced with HS, BET, and BET + AOX, while the duration of recovery time after exposure to HS could be a reason for this reduction [64]. Furthermore, BET and BET + AOX could provide sufficient energy for the body by reducing body temperature to avoid anaerobic pathways, due to lower lactic acid. The thyroid hormones have major function in the control of metabolic rate and thermogenesis in broiler [65]. The results of the present study confirmed with [65,66,67] showed that HS reduces both T_3_ and T_4_. A reduction in thyroid hormones production during HS is one of the pathways for maintenance of homeostasis [65]. While the T_3_ results under HS are consistent in most studies and have shown reduction [68,69,70], the T_4_ results are inconsistent among studies that showed reduction [71], increase [69], or no alteration [72]. While there is a correlation between ultimate muscle pH and colour, pH is not the only factor that impacts meat color [73]. In the present study there were no effects of HS on pH or colour, which disagrees with studies that argued that both cyclic and consistent HS could reduce initial and ultimate pH [74,75]. However, other studies support the present results, which showed that consistent HS [27] and short term HS have no effects on the initial and ultimate pH [76]. Furthermore, pH level reduction over time agrees with [77,78]. These studies could provide the insight that the duration of HS is an important factor that could affect pH level. From the colour aspect, the results are in line with [74], which indicated that HS has no effect on colour. Lighter breast muscles over time in BET is related to BET functioning as an osmolyte that can improve and regulate cells osmotic balance [79]. The amount of water available in meat is important, as losing water could affect the weight and correspondingly the economic value of the final product. Additionally, it may affect the appearance, the juiciness, and the tenderness of the meat. The results of the present study agree with [80,81,82,83], which indicated that HS increases water loss, which is evident in the reduced total water content, resulting in reduced cooking loss and reduced breast yield, whereas BET could improve it. It is likely that HS causes leaky membranes, resulting in loss of water from muscle cells and it would explain the reduced muscle total water content and reduced breast yield. Betaine, as an osmolyte, maintains the osmotic pressure to prevent dehydration and preserves water in cells [84]. The present results are in disagreement with [80,85], which showed that HS increased shear force; however, the results agree with [86], where HS has no impact on shear force. The duration of HS could be a reason for inconsistent results among studies.

Although no overall differences in WBSF were observed in this study, there was a reduction in the MFI in HS broilers. Texture measurements, such as WBSF, measure the force required to shear through the myofibrils and connective tissue in a standard block of meat and they are a product of both the skeletal muscle fibres and connective tissue. Alternatively, MFI is only a measure of the myofibrillar filament fragmentation. The meat tenderization process is initiated by calpain mediated proteolysis of the skeletal muscle fibre, forming smaller fragments and an increase in the MFI [87]. The results of this study agree with [74,80] that HS reduces MFI. The mechanism is that, because HS generates high amounts of reactive oxygen species, this either (i) leads to the oxidation of myofibrillar proteins that can cause protein cross-linking and toughness or (ii) leads to the direct oxidation of calpain, preventing proteolysis from occurring, or a combination of both [88]. Elsewhere, HS has been observed to increase Atrogin-1 gene expression, which is associated with muscle wasting, which would likely have consequences for meat tenderisation. Notably, BET + AOX improved MFI under HS, which may indicate that BET, in combination with AOX, provides a number of benefits to both product yield and quality.

Lipid oxidation, a process during which meat lipids oxidize and interact with other meat constituents, causes deterioration in the quality of meat and it causes undesirable effects in nutritive value. The TBARS in biological pathways, which include lipid hydroperoxides, increases in response to oxidative stress. The results are consistent with [82,89,90], whose studies indicated that HS increases the oxidative damages to muscle due to increased TBARS. Heat stress generates a high amount of reactive oxygen species due to the denaturation of proteins and cell death, which results in higher oxidative damages in tissues [82]. Moreover, it has been indicated that additives, such as BET, can improve TBARS concentration and preserve the cellular antioxidant storage under stress conditions [13,82], and there are additional effects of antioxidants [50,91] through removing ROS. In this context, the combination of dietary BET and AOX reduced meat TBARS to a greater extent than BET alone.

## 5. Conclusions

Supplemented BET conferred overall benefits, as well as improved resilience against HS in this small university study. Some additional benefits, such as increased MFI and decreased plasma lactate and muscle TBARS, were provided by supplemental BET + AOX. This study appears to show that supplemented BET is utilized by the liver, potentially to benefit important metabolic pathways, such as the methionine cycle and antioxidant production. However, this requires further investigation. Likewise, accumulation in skeletal muscle may also have a protective effect and contribute to improved growth and meat quality. The additional effects of AOX warrant further investigation, particularly with respect to shelf life and oxidation.

## Figures and Tables

**Table 1:** Effect of dietary betaine (BET) and antioxidants (AOX) on average daily gain (ADG), average daily feed intake (ADFI), and feed conversion ratio (FCR), and carcass characteristics during thermoneutral (TN) and heat stress (HS) conditions.

Temperature (T)	Thermoneutral			Heat Stress			s.e.d. ^2^	*p*-Value ^3^		
Diet	CON ^1^	BET	BET + AOX	CON ^1^	BET	BET + AOX		T	BET	Within BET
Day 0–21										
ADG, g/d	45.5	47.0	49.5	43.4	44.1	46.8	1.85	0.11	0.15	0.17
ADFI, g/d	70.8	64.3	67.3	59.7	67.9	66.3	4.35	0.50	0.88	0.87
FCR, g/g	1.56	1.37	1.36	1.38	1.55	1.42	0.098	0.70	0.46	0.49
Day 21–42										
ADG, g/d	93.3	98.9	98.0	83.3	85.2	89.3	3.09	<0.001	0.10	0.61
ADFI, g/d	182	184	197	164	171	180	7.4	0.017	0.13	0.15
FCR, g/g	1.96	1.86	2.01	1.97	2.02	2.02	0.075	0.32	0.86	0.34
Day 0–42										
ADG, g/d	69.4	72.9	73.8	63.3	64.7	68.1	1.99	<0.001	0.056	0.30
ADFI, g/d	126	124	132	112	120	123	4.8	0.033	0.21	0.24
FCR, g/g	1.82	1.70	1.79	1.76	1.85	1.81	0.055	0.35	0.82	0.69
Final weight, g	2953	3103	3138	2697	2755	2898	83.6	<0.001	0.052	0.30
Breast weight, g	672	753	785	596	640	662	24.2	<0.001	0.002	0.24

^1^ Control diet (CON) contained 50 IU/kg VitE and 0.3 mg/kg Se. ^2^ Standard error of the difference for T × CON vs pooled BET. ^3^ T—TN *vs* HS; BET—CON *vs* pooled BET treatments; Within BET—BET *vs* BET + AOX.

**Table 2:** Effect of dietary betaine (BET) and antioxidants (AOX) on acid base balance and blood gas during thermoneutral (TN) and heat stress (HS) conditions.

Temperature (T)	Thermoneutral			Heat Stress			s.e.d. ^2^	*p*-Value ^3^		
Diet	CON ^1^	BET	BET + AOX	CON ^1^	BET	BET + AOX		T	BET	Within BET
pH	7.31	7.31	7.32	7.41	7.37	7.38	0.037	0.021	0.64	0.84
pCO_2_, mm Hg	54.1	51.9	54.1	40.5	42.7	41.4	4.13	0.002	0.96	0.92
Total CO_2_, mM	27.1	27.7	32.1	26.0	27.2	28.4	1.39	0.14	0.067	0.058
pO_2_, mm Hg	31.3	34.0	29.6	35.1	31.9	33.9	1.61	0.15	0.54	0.47
O_2_ saturation, %	49.2	58.8	56.8	62.0	64.0	67.1	4.88	0.029	0.17	0.91
HCO_3_, mM	27.3	26.5	30.4	24.7	25.9	26.8	1.14	0.024	0.17	0.050
Anion gap, mM	19.3	16.0	15.8	16.3	15.5	13.3	1.36	0.089	0.039	0.37
Base excess, mM	−1.38	−0.23	1.90	−0.10	0.93	1.78	0.994	0.36	0.047	0.15

^1^ Control diet (CON) contained 50 IU/kg VitE and 0.3 mg/kg Se. ^2^ Standard error of the difference for T × CON vs pooled BET. ^3^ T—TN *vs* HS; BET—CON *vs* pooled BET treatments; Within BET—BET *vs* BET + AOX.

**Table 3:** Effect of dietary betaine (BET) and antioxidants (AOX) on haematology, electrolytes, metabolites and plasma thyroid hormones during thermoneutral (TN) and heat stress (HS) conditions.

Temperature (T)	Thermoneutral			Heat Stress			s.e.d. ^2^	*p*-Value ^3^		
Diet	CON ^1^	BET	BET + AOX	CON ^1^	BET	BET + AOX		T	BET	Within BET
Haematocrit, %	21.5	21.8	22.0	18.8	18.0	19.8	1.04	0.003	0.78	0.35
Hgb^4^, g/dL	6.93	7.38	7.45	6.38	6.20	6.83	0.312	0.006	0.26	0.28
Potassium, mM	6.38	5.80	5.80	5.73	5.83	5.30	0.239	0.071	0.092	0.29
Sodium, mM	150	149	150	149	150	149	1.3	0.82	0.96	0.93
Chloride, mM	112	112	110	114	115	114	1.3	0.014	0.82	0.26
Calcium, mM	1.48	1.46	1.49	1.36	1.43	1.42	0.037	0.034	0.42	0.82
Lactate, mM	9.17	7.89	5.24	5.53	5.17	3.73	0.74	<0.001	0.010	0.012
Glucose, mM	15.6	14.5	14.8	15.2	15.7	15.5	0.61	0.30	0.56	0.92
T_3_^4,5^, *p*g/mL	4.76	6.47	6.08	5.16	3.76	3.43	0.977	0.008	0.97	0.61
T_4_^4^, *p*g/mL	4.57	5.54	5.74	3.23	3.16	4.49	1.366	0.049	0.33	0.78

^1^ Control diet (CON) contained 50 IU/kg VitE and 0.3 mg/kg Se. ^2^ Standard error of the difference for T × CON *vs* pooled BET. ^3^ T—TN *vs* HS; BET—CON *vs* pooled BET treatments; Within BET—BET *vs* BET + AOX. ^4^ Haemoglobin (Hgb); triiodothyronine (T_3_); Thyroxine (T_4_). ^5^ T × BET interaction (*p* = 0.018).

**Table 4:** Effect of dietary betaine (BET) and antioxidants (AOX) and time on meat colour and pH during thermoneutral (TN) and heat stress (HS) conditions. Since there were no main or interactive effects of HS the data have been pooled across temperature regimes.

		Diet			s.e.d. ^2^	*p*-Value ^3^				
Time/Diet	Hours	CON^1^	BET	BET + AOX		Time	BET	Within BET	Timex BET	Time × within BET
L*	24	54.3	55.1	54.3	0.967	0.011	0.31	0.52	0.061	0.82
	96	54.2	56.0	55.4						
a*	24	2.20	2.50	2.30	0.283	0.91	0.68	0.57	0.26	0.82
	96	2.22	2.31	2.40						
b*	24	1.43	1.59	1.65	0.268	0.062	0.97	0.49	0.77	0.82
	96	1.74	1.82	1.88						
pH	0.25	6.68	6.68	6.78	0.053	<0.001	0.27	0.094	0.67	0.10
	1	6.48	6.49	6.58						
	24	5.92	5.93	5.93						

^1^ Control diet (CON) contained 50 IU/kg VitE and 0.3 mg/kg Se. ^2^ Standard error of the difference for Time × CON *vs* pooled BET. ^3^ Time—24 *vs* 96 or 0.25 *vs* 1 *vs* 24 h; BET—CON *vs* pooled BET treatments; Within BET—BET *vs* BET + AOX.

**Table 5:** Effect of dietary betaine (BET) and antioxidants (AOX) on chicken breast objective eating quality measures during thermonneutral (TN) and heat stress (HS) conditions.

Temperature (T)	Thermoneutral			Heat Stress			s.e.d. ^2^	*p*-Value ^3^		
Diet	CON ^1^	BET	BET + AOX	CON ^1^	BET	BET + AOX		T	BET	Within BET
Cooking loss^4^, %	27.5	27.4	26.9	23.0	25.8	26.0	1.27	0.007	0.21	0.84
Drip loss^5^, %	2.11	1.74	1.78	3.43	1.15	2.22	0.51	0.33	0.007	0.20
Water content, %	74.8	76.5	75.9	74.6	75.2	75.3	0.79	0.079	0.13	0.56
Shear force^6^, N	18.4	20.4	23.9	23.0	22.8	21.3	1.95	0.30	0.24	0.34
Breast yield, %	22.8	24.4	24.9	22.7	22.9	23.6	0.92	0.057	0.066	0.37
MFI^7^	86.3	79.7	87.7	58.1	62.8	81.6	6.23	<0.001	0.14	0.003
0.5 h TBARS^8^	0.21	0.20	0.17	0.21	0.21	0.17	0.040	0.88	0.37	0.20
24 h TBARS^8,9^	5.32	3.48	2.00	6.12	5.51	5.32	0.545	<0.001	<0.001	0.029

^1^ Control diet (CON) contained 50 IU/kg Vit E and 0.3 mg/kg Se. ^2^ Standard error of the difference for T × CON *vs* pooled BET. ^3^ T—TN *vs* HS; BET—CON *vs* pooled BET treatments; Within BET—BET *vs* BET + AOX. ^4^ T × BET interaction (*p* = 0.075). ^5^ T × BET interaction (*p* = 0.052). ^6^ T × BET (*p* = 0.10) and T × within BET (*p* = 0.087) interactions. ^7^ Myofibrillar fragmentation index (MFI); T × BET interaction (*p* = 0.031). ^8^ Thiobarbituric acid reactive substances (TBARS) in mg/kg of malondialdehyde (MDA). ^9^ T × BET (*p* = 0.008) and T × within BET (*p* = 0.10) interactions.

**Table 6:** Effect of dietary betaine (BET) and antioxidants (AOX) on plasma and tissue betaine concentrations during thermonneutral (TN) and heat stress (HS) conditions.

Temperature (T)	Thermoneutral			Heat Stress			s.e.d. ^2^	*p*-Value ^3^		
Diet	CON ^1^	BET	BET + AOX	CON ^1^	BET	BET + AOX		T	BET	Within BET
Plasma ^4^, µmol/L	85.0	140	145	102	109	125	10.50	0.043	<0.001	0.14
Muscle ^5^, µmol/g	321	635	413	425	518	567	9.38	0.43	0.009	0.16
Liver ^6^, µmol/g	403	191	187	208	143	145	45.1	<0.001	<0.001	0.98

^1^ Control diet (CON) contained 50 IU/kg Vit E and 0.3 mg/kg Se. ^2^ Standard error of the difference for T × CON *vs* pooled BET. ^3^ T—TN *vs* HS; BET—CON *vs* pooled BET treatments; Within BET—BET *vs* BET + AOX. ^4^ T × BET interaction (*p* = 0.002). ^5^ T × within BET interaction (*p* = 0.050). ^6^ T × BET interaction (*p* = 0.002).
